# Anesthetic Management of a Patient With Massive Pulmonary Secretion During Cardiopulmonary Bypass Probably Due to Transfusion-Related Acute Lung Injury Type Ⅱ

**DOI:** 10.7759/cureus.37405

**Published:** 2023-04-10

**Authors:** Yasuhiro Watanabe, Mitsumasa Miyagi, Toru Kaneda

**Affiliations:** 1 Department of Anesthesia, Japanese Red Cross Shizuoka Hospital, Shizuoka, JPN

**Keywords:** hyperpermeability edema, endotracheal effusion, cardiopulmonary bypass, transfusion related acute lung injury, packed red blood cell transfusion

## Abstract

Transfusion-related acute lung injury (TRALI) is potentially life-threatening adverse reaction associated with blood transfusion and can induce perioperative pulmonary secretion. TRALI that develops during cardiopulmonary bypass (CPB) may be difficult to detect; however, the pathophysiology might manifest as derangements in CPB operations. A 79-year-old man was scheduled to undergo partial replacement of the aortic arch with CPB. Two units of red blood cells were loaded into the priming solution. Although the vital signs, including oxygenation, remained stable in the prebypass period, perfusionists noticed a decreasing trend in the venous reservoir level early in the CPB operations. The trend continued even during circulatory arrest with selective cerebral perfusion, resulting in the termination of the modified hemofiltration. Surgical procedures were accomplished uneventfully; however, a large amount of fluid was required to maintain the minimal reservoir level and CPB flow. The total fluid balance during CPB was +8,233 mL, which was quite unusual in our practice. When 800 mL of massive pulmonary secretion was detected before CPB withdrawal, the etiology could not be determined simultaneously; nonetheless, systemic vascular hyperpermeability was speculated to be the underlying pathophysiology. Our therapeutic approach following the treatment of acute respiratory distress syndrome contributed to halting the deterioration of lung injury. Although the pneumothorax developed on the first postoperative day, the patient was treated with the insertion of a chest drainage tube. Subsequently, the patient had a good course and was discharged without respiratory complications. In conclusion, massive pulmonary secretion, probably due to TRALI type II, was associated with derangements in CPB operations. Prompt identification of the underlying pathophysiology and appropriate intervention is crucial.

## Introduction

Transfusion-related acute lung injury (TRALI) is a potentially fatal adverse reaction and the incidence is 1.5 reactions per 100,000 components transfused [1]. TRALI occurs during or within 6 h of blood transfusion and is defined as acute onset hypoxemia accompanied by bilateral pulmonary edema without evidence of left atrial hypertension [2]. TRALI type II, formerly referred to as “possible TRALI,” represents acute lung injury in a transfused patient with other risk factors, such as cardiac surgery, for developing acute respiratory distress syndrome (ARDS) [2]. Intraoperatively, TRALI can be detected by pulmonary secretion accompanied by acute onset of hypoxemia i.e., P/F ratio (ratio of partial pressure of arterial oxygen (PaO_2_) to fraction of inspired oxygen (FiO_2_) ≤ 300 or SpO_2_ (oxygen saturation measured by pulse oximetry) < 90% on room air [2]. However, detection may be difficult in cases where it occurs during cardiopulmonary bypass (CPB) due to the lack of sufficient information. Although TRALI occurrence during cardiac surgery has been reported [3,4], few clinical reports have addressed cases of TRALI associated with derangements in CPB operations. Here, we report a case of massive pulmonary secretions during CPB, probably induced by TRALI type II. The patient was successfully managed with a pathophysiology-based therapeutic approach and discharged without serious complications.

## Case presentation

**Figure 1:**
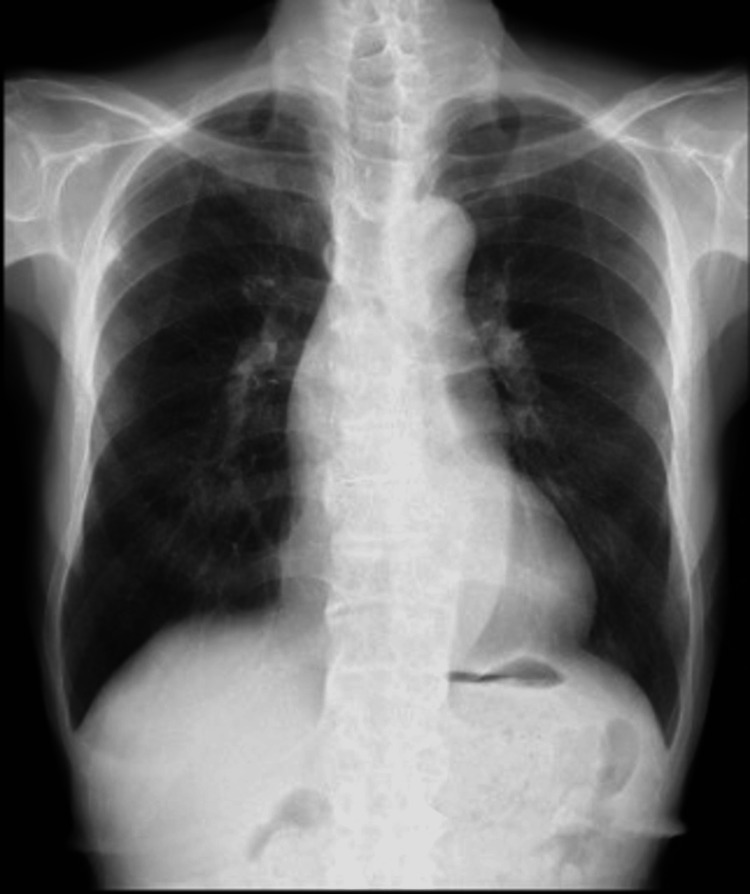
Preoperative chest radiograph. Clear lung fields with a cardiothoracic ratio of 48.0% were observed in the upright position.

A 79-year-old man (height, 174.5 cm; weight, 49.6 kg; body surface area, 1.59 m^2^; EuroSCORE II, 8.43%) presented with a thoracic aortic aneurysm and aortic regurgitation (AR). The patient had undergone percutaneous transluminal coronary angioplasty for myocardial infarction eight years previously and was prescribed carvedilol (2.5 mg), aspirin (100 mg), and warfarin (2 mg) for the prevention of intracardiac thrombus formation. Additionally, the patient was prescribed enalapril maleate (2.5 mg) and furosemide (20 mg) for hypertension treatment. The patient was prescribed dapagliflozin propylene glycolate hydrate (10 mg), linagliptin (5 mg), and metformin hydrochloride (200 mg) for the treatment of diabetes mellitus. The patient had no history of anaphylactic reactions. He had never smoked, and preoperative chest radiographs demonstrated clear lung fields with a cardiothoracic ratio (CTR) of 48.0% and no congestion (Figure 1).

Electrocardiography showed sinus rhythm with abnormal Q waves in leads II, III, aVF, and V1−V5. Transthoracic echocardiography revealed akinesis of the left anteroseptal wall and apex with an ejection fraction of 45%, mild AR, and no intracardiac thrombi. Coronary angiography showed 50% stenosis of the right coronary artery (RCA #3), chronic total occlusion of the left anterior descending artery (LAD #7 proximal) with collateral flow from the RCA, and no stenotic lesion in the left circumflex artery with drug-eluting stents implanted. Consequently, partial replacement of the aortic arch using an extracorporeal circuit (CAPIOX^®️^ FX25, TERUMO Corporation, Tokyo, Japan) was planned. Warfarin was discontinued two days prior to surgery. The results of blood tests taken the day before surgery showed a prolongation of the international normalized ratio of prothrombin time; therefore, menatetrenone (10 mg) was administered to antagonize the residual effects of warfarin. The results also showed anemia, low platelet count, and renal dysfunction, as well as high glycated hemoglobin and N-terminal pro-brain natriuretic peptide levels. 

General anesthesia was induced with midazolam (0.5 mg) and sevoflurane inhalation after administering fentanyl (300 μg) and remifentanil 0.05 μg∙kg^-1^∙min^-1^. Muscle relaxation was achieved with rocuronium (50 mg), and the trachea was intubated without aspiration. Transesophageal echocardiography (TEE) was performed, followed by insertion of a central venous catheter and a pulmonary artery catheter. Subsequently, cardiac output, mixed venous oxygen saturation (SvO_2_), central venous pressure (CVP), and pulmonary arterial pressure (PAP) were monitored continuously (Vigilance Ⅱ^TM^, Edwards Lifesciences, Irvine, CA, USA). Anesthesia was maintained with sevoflurane, continuous infusion of remifentanil (0.05−0.3 μg∙kg^-1^∙min^-1^), and fentanyl. Nicorandil (0.05 mg∙kg^-1^∙hr^-1^) and carperitide (0.01 μg∙kg^-1^∙min^-1^) were administered, and tranexamic acid (300 mg) was administered to suppress hyperfibrinolysis. Until CPB was established, systolic blood pressure was maintained around 90 mmHg with norepinephrine infusion fixed at 0.01 μg∙kg^-1^∙min^-1^ and several phenylephrine boluses. Other vital signs, such as heart rate, SpO_2_, airway pressure, CVP, PAP, and SvO_2_, remained stable. Systolic and diastolic PAP ranged between 12−16 mmHg and 6−8 mmHg, respectively, with a CVP of 6−8 mmHg. Prebypass fluid administration was 600 mL of crystalloid, including 500 mL of bicarbonate Ringer’s solution and 100 mL of normal saline containing cefazolin sodium (1 g), whereas the urine output was 120 mL. After administering 17,000 units of heparin, an arterial cannula was inserted into the ascending aorta without aortic dissection. Subsequently, maintenance of anesthesia was switched to a continuous infusion of propofol (2−3 mg∙kg^-1^∙h^-1^). The CPB priming solutions are shown in Table 1.

**Table 1:** Cardiopulmonary bypass priming solution. In our practice, the priming solution contains methylprednisolone sodium succinate 1 g. Two units of red blood cells were loaded to avoid excessive hemodilution.

Methylprednisolone sodium succinate (1 g) in normal saline (mL)	16
Cefazolin sodium (1 g) in normal saline (mL)	10
Heparin (1,000 units/mL)	3
Furosemide (10 mg/mL)	2
Hydroxyethyl starch (6% HES 130000) (mL)	500
Bicarbonate Ringer's solution (mL)	300
Red blood cells (mL)	280
20% Mannitol (mL)	200
Total volume (mL)	1,311

Blood tests performed after heparinization demonstrated good oxygenation (Table 2, column 1). A dual-stage cannula was inserted under TEE monitoring, and CPB was initiated with spontaneous beating retention. After a full flow (2.4 L∙m^-2^∙min^-1^) was achieved one minute later, mechanical ventilation was terminated, and a positive end-expiratory pressure (PEEP) of 3 cmH_2_O was applied to the respiratory circuit. After another 3 min, a venting catheter was inserted via the right upper pulmonary vein into the left ventricle, and systemic cooling was initiated. The sinus rhythm of the heart became ventricular fibrillation 11 min after the initiation of cooling (i.e., 15 min after establishing CPB). The perfusionists began to notice unusual abnormalities during CPB, which was a depletion in the venous reservoir level. In the first 30 min, 2,100 mL of crystalloid and 500 mL of 6% hydroxyethyl starch, except for the priming solution, were administered. Since the decompression of the right heart system was confirmed, we attributed the etiology to dilation of resistance arteries and subsequent enlargement of vascular beds. Strikingly, in the next 30 min, additional 3,400 mL of crystalloid and 500 mL of hydroxyethyl starch, along with 280 mL of red blood cells (RBCs) were administered to maintain the minimum reservoir level of 300 mL. Fifty-eight min after the initiation of cooling, the bladder temperature stabilized at 26 ℃, and circulatory arrest was induced, followed by cross-clamping of the ascending aorta.

**Table 2:** The results of arterial blood gas analysis. (Column 1) Arterial blood gas analysis performed after heparinization demonstrated good oxygenation. (Column 2) Blood tests performed after the administration of protamine showed deteriorated oxygenation, indicating acute lung injury. (Column 3) Blood tests were performed after sternum closure. Our therapeutic approach contributed to halting the further deterioration of acute lung injury. (Column 4)  Blood tests were performed on the first postoperative day. Despite the onset of pneumothorax, oxygenation returned to the normal range. PaCO_2_: Partial pressure of arterial carbon dioxide; PaO_2_: Partial pressure of arterial oxygen; FiO_2_: Fraction of inspired oxygen; P/F ratio: Ratio of partial pressure of arterial oxygen to fraction of inspired oxygen.

	1	2	3	4	Reference value
pH	7.437	7.338	7.363	7.445	7.35 − 7.45
PaCO_2 _(mmHg)	35.2	39.7	38.8	37.4	32 − 45
PaO_2_ (mmHg)	223	270	140	190	80 − 100
FiO_2_	0.4	1.0	0.47	0.45	0.21
P/F ratio	544	270	298	422	≥ 400
Hemoglobin (g/dL)	10.1	6.8	10.1	11.8	13.7 − 16.8
Hematocrit (%)	31.3	21.2	31.4	35.3	40.7 − 50.1
Base excess (mmol/L)	−0.4	−4.1	−3.0	1.8	−3.2 − 2.3
Lactate (mmol/L)	0.5	1.3	1.1	1.8	0.5 − 1.6
Blood glucose (mg/dL)	78	117	146	153	70 − 105

Cardiac arrest was introduced via antegrade infusion of cardioplegia and maintained with continuous retrograde and intermittent antegrade perfusion. After insulating the brachiocephalic artery (BCA) and the left common carotid artery (LCCA), the aortic arch was dissected between the LCCA and the left subclavian artery. Antegrade selective cerebral perfusion was established, and modified ultrafiltration (MUF; CAPIOX^®️^ Hemoconcentrator 11S, TERUMO Corporation, Tokyo, Japan) was initiated. After the distal anastomosis of the vascular prosthesis (J Graft SHIELD NEO^®️^, Japan Lifeline, Tokyo, Japan) was completed, systemic circulation was resumed via side branch perfusion, and rewarming was initiated. Circulatory arrest time was 43 min. Proximal anastomosis of the graft, cannulation of the venting catheter into the graft, and aortic de-clamping were sequentially performed. Finally, reconstruction of the BCA and LCCA was accomplished uneventfully, during which a single defibrillation restored the sinus rhythm at an energy of 20 J. Aortic cross-clamping and selective cerebral perfusion durations were 76 and 113 min, respectively. The lungs were manually ventilated to remove the left ventricular venting catheter, and the anesthesiologist felt resistance and found massive secretion floating in the endotracheal tube. The fluid was non-bloody serosanguineous and was counted to be 800 mL in a single suction drainage. Afterward, repeated intermittent drainage was required, resulting in a total volume of 950 mL at the end of surgery. Skin lesions suggesting anaphylactic reactions were not found as far as can be observed. Additional methylprednisolone sodium succinate 125 mg was given as a bolus, and a continuous infusion of sivelestat sodium hydrate (0.2 mg∙kg^-1^∙hr^-1^) was conducted for 24 h. The same dose of methylprednisolone was administered once postoperatively and twice on the first postoperative day.

Mechanical ventilation was resumed by pressure-controlled ventilation (FiO_2_ of 1.0, PEEP of 10 cmH_2_O, peak inspiratory pressure of 21 cmH_2_O). Under infusion of dobutamine 3 μg∙kg^-1^∙min^-1^ and norepinephrine 0.2 μg∙kg^-1^∙min^-1^, TEE demonstrated cardiac contractility comparable with that of the prebypass period. However, an additive infusion of vasopressin (2 units/h) was required to raise the systolic blood pressure and allow withdrawal from CPB. Although pulmonary capillary wedge pressure was not measured, diastolic PAP and CVP immediately after CPB withdrawal were 15−20 and 8−9 mmHg, respectively, with a high dose of vasopressors in the Trendelenburg position. Blood tests after administration of protamine showed the deteriorated oxygenation (Table 2, column 2). The CPB time was 243 min, and surprisingly, the total fluid balance during CPB increased to +8,233 mL (In: 15,089 mL including six units of RBCs, Out: blood loss, 145 mL; urine output, 2,020 mL; water removal by MUF, 2,130 mL; residual blood in the CPB circuit, 1,761 mL; pulmonary secretion, 800 mL). After CPB withdrawal, ten units of RBCs, 12 units of fresh frozen plasma, 20 units of platelet concentrate, and 781 mL of salvaged blood were administered to facilitate hemostasis and hemodynamic stabilization. There was no further deterioration of oxygenation, and administration of vasopressin and norepinephrine was discontinued until the end of surgery. Blood tests performed after sternum closure are shown in Table 2, column 3.

**Figure 2:**
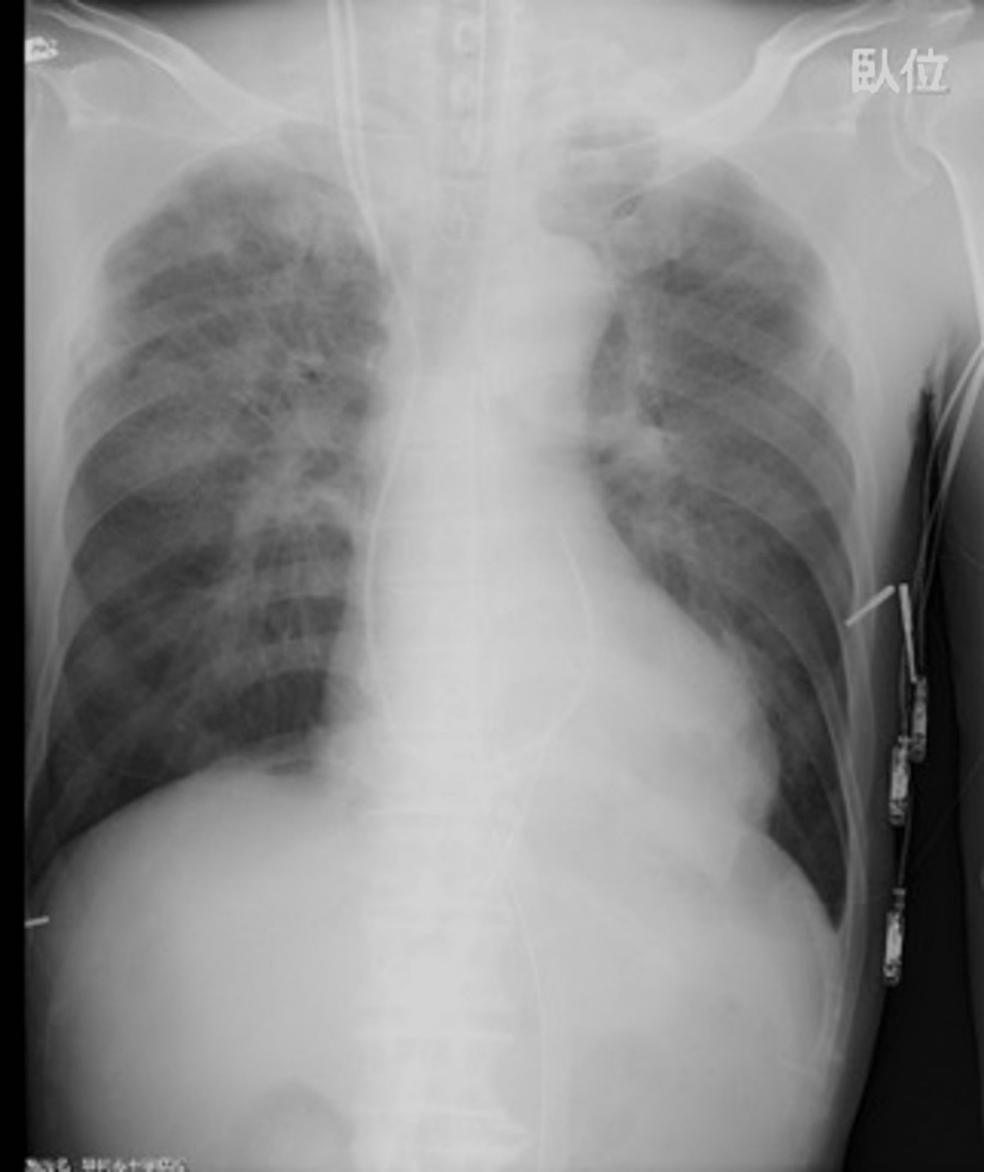
Postoperative chest radiograph. Bilateral pulmonary edema and disproportionately mild cardiomegaly with a cardiothoracic ratio of 53.5% were observed in the supine position.

The operation duration and total anesthesia time were 487 and 574 min, respectively, and the intraoperative total fluid balance resulted in +8,574 mL. Chest radiograph immediately after surgery revealed bilateral pulmonary edema without marked cardiomegaly (Figure 2).

**Figure 3:**
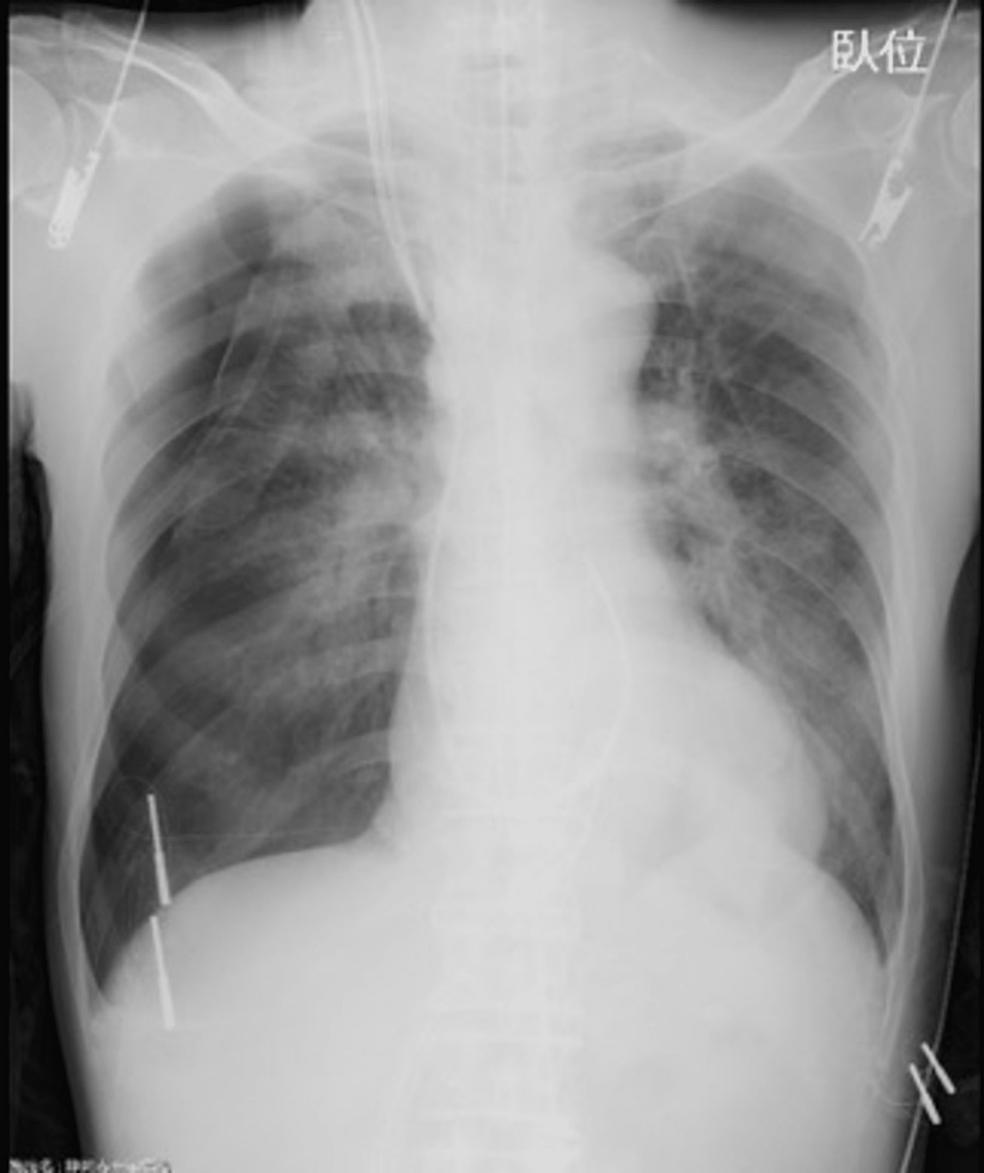
Chest radiograph on the first postoperative day. Right pneumothorax with a cardiothoracic ratio of 50.5% was revealed.

In the intensive care unit, although the pulmonary secretion decreased considerably, intermittent suctioning was required. On the first postoperative day, the chest radiograph revealed a right pneumothorax, which was treated by inserting a drainage tube (Figure 3).

**Figure 4:**
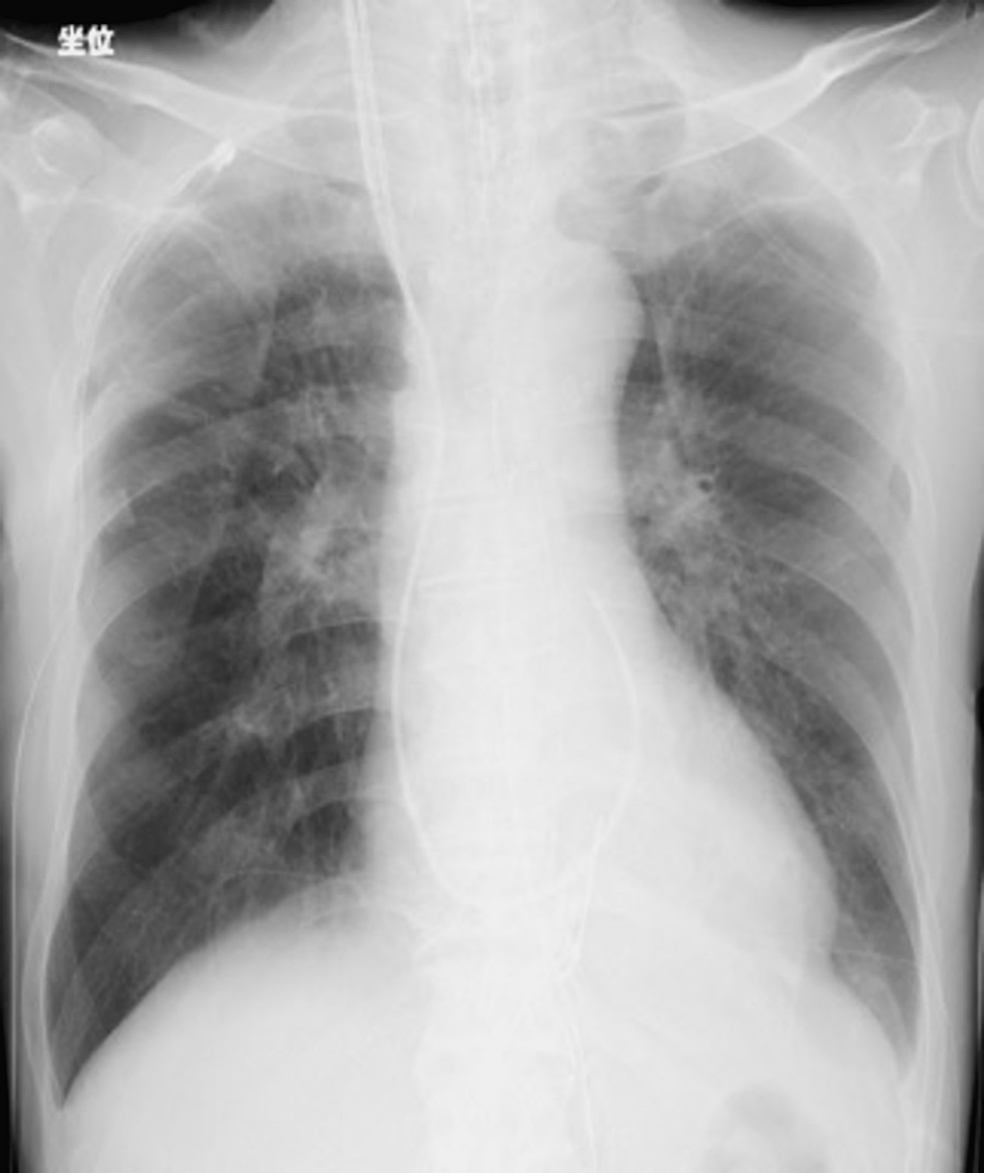
Chest radiograph on the second postoperative day. Improvement in both right pneumothorax and bilateral pulmonary edema was confirmed.

Nevertheless, oxygenation returned to the normal range (Table 2, column 4), and pulmonary secretion was rarely aspirated. On the second postoperative day, the chest radiograph revealed improvement in both pneumothorax and bilateral pulmonary edema, and the patient was extubated (Figure 4).

Afterward, the patient had a relatively good postoperative course and was discharged 24 days postoperatively without respiratory or cardiac complications.

## Discussion

We highlighted the significance of pathophysiology-oriented anesthetic management in a patient with massive pulmonary secretion probably due to TRALI type Ⅱ. Although it was difficult to determine the etiology immediately, given the derangements in CPB operations, systemic vascular hyperpermeability was considered the underlying pathology. We believe our therapeutic approach has helped to halt the deterioration of acute lung injury and prevent fatal outcomes.

Pulmonary edema can be categorized into cardiogenic (hydrostatic) and non-cardiogenic (permeability) pulmonary edema [5]. The etiology of cardiogenic edema is congestive heart failure as the underlying mechanism. In contrast, the pathogenesis of non-cardiogenic edema is miscellaneous, particularly in cardiac surgery, and includes anaphylaxis, CPB-related lung injury, protamine reaction, reexpansion pulmonary edema, negative pressure pulmonary edema, and TRALI. In the present case, although AR had been detected preoperatively, antegrade cardioplegia administration promptly achieved initial cardiac arrest, supporting the mild degree of regurgitation. Furthermore, the left ventricular venting catheter was inserted with certainty using TEE, and the surgeons never observed dilatation of the left heart system. On the other hand, a dual-stage cannula for venous drainage was also placed under TEE monitoring, and effective gravity venous drainage and decompression of the right heart system were confirmed early in the CPB operations. However, retrospective investigation of anesthetic records revealed that in the first 15 min after CPB initiation, coinciding with the elapsed time before the sinus rhythm of the heart became ventricular fibrillation, both a pulse pressure of 10−15 mmHg in systemic blood pressure and PAP of 3−5 mmHg were retained, indicating the presence of antegrade perfusion via the pulmonary vasculature. Even after the electrocardiogram demonstrated ventricular fibrillation, there might have been much less pulmonary flow; nevertheless, it is difficult to conclude that the pulmonary blood flow *per se* led to massive pulmonary secretion. Vital signs, including respiratory status, remained stable during the pre bypass period. Blood tests after heparinization showed the P/F ratio over 500, and there was no evidence of new wall motion abnormality, new valvulopathy, or right heart failure throughout the surgery. Postoperative radiographs showed bilateral pulmonary edema with disproportionately mild cardiomegaly (Figure 2), considering that the preoperative radiograph was obtained in the upright position (Figure 1), suggesting non-cardiogenic edema. Perfusionists noticed an unusual depletion of the venous reservoir level early in the CBP operations. Practically, within the first 60 min, 6,780 mL of fluid, including two units of RBCs, except for the priming solution, was administered to maintain the minimum reservoir level. It was also quite unusual for MUF, initiated after inducing circulatory arrest, to be discontinued because of a decreasing trend in the reservoir level, despite that a large amount of fluid had been administered until then. Consequently, the fluid balance during CPB increased to +8,233 mL, and on the other hand, RBCs transfused during CPB operation (six units in total, including two units loaded in the priming solution) to maintain the hematocrit level of 23% seem disproportionately low. These findings suggest systemic vascular hyperpermeability rather than systemic vasodilation as an underlying pathophysiology. 

Based on these retrospective analyses, we concluded that both massive pulmonary secretion and derangements in CPB operations were derived from TRALI type II and accompanying systemic vascular hyperpermeability developed by RBCs transfused from the CPB circuit. Furthermore, given the time course, the RBCs loaded in the priming solution are likely to be the initiator and retained antegrade pulmonary flow even after CPB initiation plays a vital role in pulmonary edema formation. Strictly speaking, TRALI cannot be diagnosed without the confirmation of hypoxemia and the exclusion of left atrial hypertension [2]; however, it is impossible under CPB. Although the role of corticosteroid in ARDS management is still controversial [6], we believe that the administration of methylprednisolone is reasonable because the systemic inflammation, the cardinal pathology of TRALI as the cause of ARDS [7,8], is considered to be the underlying pathophysiology. Importantly, a different therapeutic approach (e.g., diuretics) based on inappropriate speculation may have led to a more serious outcome. The two-hit model proposes that the development of TRALI generally requires preexisting clinical conditions (i.e., the first hit) before blood transfusion [9]. In the present case, the first hit can be represented by surgical insult, mechanical ventilation, and extracorporeal circulation, all of which induce inflammation. The development of ARDS is often multifactorial in cardiac surgery patients [10]; indeed, multiple transfusions have been identified as an independent risk factor [11,12]. On the other hand, the molecular basis of CPB-related lung injury is systemic inflammatory response, including longitudinal activation of the complement system and proinflammatory cytokines, production of reactive oxygen species, as well as neutrophil sequestration in the lung [13,14]. Specifically, the neutrophil also plays a key role in the pathogenesis of TRALI [15]. In cardiac surgery using CPB, ARDS was diagnosed 2−20 days postoperatively in patients with no other explainable pathology [12]. Here, given the time course, it seems still difficult to ascribe both the derangements in CPB operations and massive pulmonary secretion to CPB-induced lung injury itself. Although serological tests for transfused blood products were not performed, TRALI remains clinically diagnosed and does not require the detection of cognate white blood cell antibodies [2]. Diagnostically, it remains challenging to distinguish anaphylaxis as the underlying pathophysiology in this case. Theoretically, all extracorporeal devices and all components in the priming solution could be the trigger. Both hydroxyethyl starch and mannitol preparations can induce anaphylactic reactions [16,17]; however, the incidence is quite low [18], and skin symptoms, such as erythema and wheals, were not detected at all perioperatively.

## Conclusions

Massive pulmonary secretion during CPB, probably due to TRALI type II, was related to derangements in CPB operations. Although a definite diagnosis was not immediately determined, our therapeutic approach has contributed to eliminating the exacerbation of acute lung injury. Hence, identifying the underlying pathophysiology and appropriate intervention is critical when a massive pulmonary secretion is encountered during CPB.
